# Low Medication Knowledge and Adherence to Oral Chronic Medications among Patients Attending Community Pharmacies: A Cross-Sectional Study in a Low-Income Country

**DOI:** 10.1155/2020/4392058

**Published:** 2020-01-11

**Authors:** Gashaw Binega Mekonnen, Dessalegn Asmelashe Gelayee

**Affiliations:** ^**1**^ Department of Clinical Pharmacy, School of Pharmacy, College of Medicine and Health Sciences, University of Gondar, Gondar, Ethiopia; ^2^Department of Pharmacology, School of Pharmacy, College of Medicine and Health Sciences, University of Gondar, Gondar, Ethiopia

## Abstract

**Objective:**

To investigate the level of knowledge, medication adherence, and the relationship among patients taking chronic oral medication and attending community pharmacies in Gondar, Northwest Ethiopia.

**Methods:**

A cross-sectional study was conducted among adult chronic disease patients who were taking oral medications and getting service in 19 community pharmacies in Gondar City, Northwest Ethiopia. Patients' medication knowledge and adherence were assessed using interviewer-administered validated questionnaires.

**Results:**

Of the 402 study participants (mean ± SD age = 44.7 ± 16.9 years, range = 18–86 years), 53.2% were males, 84.8% were aged <65 years, and 60.7% had high school education and above. About 348 (88.6%) respondents have used oral medications for more than one year. Less than half of respondents have good medication knowledge (*n* = 54, 38.3%) and were adherent to their medication (*n* = 158, 39.3%). Chronic disease patients with high school and above education are 5.35 times (95% CI: 3.231–8.857, *p* < 0.001) more likely to have good knowledge of their medication and having good medication knowledge was linked to higher medication adherence (AOR, 95% CI = 10.300 [6.16517.209]; *p* < 0.001). A statistically significant correlation was observed between the scores of medication knowledge and adherence (*r* = −0.471, *p* < 0.001).

**Conclusion:**

The majority of patients on oral chronic medications and attending community pharmacies in Gondar town do not have good medication knowledge and were nonadherent. Community pharmacists need to engage in medication counseling to improve medication knowledge and adherence of chronic disease patients.

## 1. Introduction

Adherence to chronic medications is one of the key factors in achieving therapeutic success. Patient-specific education and counseling are necessary to attain the desired medication adherence in geriatric patients or patients with chronic diseases. Medication adherence can be influenced by many factors, including the communication between patients and health professionals, the patient's knowledge and beliefs, and the patient's cognitive, psychological, and financial support [1–4]. A patient's knowledge about their medication can influence their trust in the medication's use and influence their adherence. Medication knowledge can be defined as the awareness of a drug's name, purpose, special instructions and dosing schedule, adverse effects, and necessary dietary modifications [5, 6]. However, asking some simple questions to the patients, such as what the medication is for, when it should be taken, and how it should be taken, can inform the researcher about their knowledge status, drug-taking behavior, and medication adherence.

Adherence to medication ranges from 38 to 57% and approximately half of the patients, high especially in those suffering from chronic diseases, are nonadherent to medications [7–9]. Nonadherence contributes to adverse drug events, unplanned hospitalization, and increased emergency visits especially in older individuals [7]. As a result, more than 30% of the medicine-related hospital admissions occur [10, 11] due to the fact that poor adherence to the cardiovascular system medications such as angiotensin-converting enzyme inhibitors, beta-blockers, and statins, for chronic coronary artery disease, was associated with a 10% to 40% relative risk increase of hospitalizations and a 50% to 80% relative risk of mortality [12]. Nonadherence to statins in the year after hospitalization for myocardial infarction was associated with a 12% to 25% increase in the relative hazard for mortality [13].

Improving the quality of pharmaceutical services through increasing access to medicine and promoting the appropriate use is among the priority policy directions in Ethiopia. Pharmacy service is an essential component of health care delivery in Ethiopia [14]. The pharmacy professional provided pharmacy service in public health institutions or community drug retail outlets. In developing countries, the community drug retail outlet is the major source of medicines because of the ease of access, wider availability of medicines, less waiting time, and longer working hours [15]. In Ethiopia, community drug retail outlets are divided into pharmacies, drug stores, and rural drug vendors on top of the qualification of dispensers and the type of medications they are supposed to dispense. Pharmacies are a place where medicines are dispensed only by pharmacists (with a qualification of a university degree or above), drug shops by druggists (with a qualification of diploma in pharmacy), and rural drug vendors by health assistants [16].

Community pharmacy is expected to give support by providing appropriate, understandable, and relevant information to the patient for their medication and to help physicians rationalize their prescription [17]. However, different studies conducted in Ethiopia revealed that community pharmacists are not adequately providing medication counseling and monitoring and describing the long-term benefits of medication adherence to chronic diseases because of the lack of medication knowledge, lack of updated drug information, high patient load, absence of private counseling room, and underestimation of the importance of counseling. These lead to poor patient knowledge and low medication adherence [18–21].

A patient's poor knowledge of prescribed drugs and lower medication adherence can lead to a burden on public health care. Chronic conditions such as high blood pressure, diabetes, and asthma require a partnership between health care providers and patients to achieve long-term therapeutic and mortality outcome goals. To the best of our knowledge, no studies have assessed medication knowledge and adherence among patients under chronic oral medication treatment in Gondar town. Thus, adherence to oral chronic medication as a treatment in adult people in this region is poorly understood, and the most crucial factors for improving adherence are not yet fully investigated. The goal of the present investigation is to assess the level of knowledge and medication adherence in patients under oral chronic medication treatment in community pharmacy settings in Gondar, Northwest Ethiopia. Moreover, it aims to examine the possible associations between these two factors, together with the patient's sociodemographic and personal data. Hopefully, understanding which factors affect the level of medication knowledge and adherence will be useful in devising strategies that could ultimately lead to more positive clinical outcomes and decreased health care costs.

## 2. Methods

### 2.1. Study Setting and Design

This cross-sectional study was conducted at 19 community pharmacies working in Gondar town from January 1 to March 30, 2017. The town is located about 727 km away from Addis Ababa, Ethiopia. According to the 2011 Central Statistical Agency of Ethiopia report, the town has a total population of 227,100. There are 19 pharmacies and 33 drug stores currently serving people living in and around Gondar [14].

### 2.2. Sample Size Determination and Sampling Procedure

The sample size was determined using a single proportion formula of *n*=*z*^2^*p*(1 − *p*)/*w*^2^ and 5% nonresponse with the following assumptions: a *z* value (at 95% level of significance = 1.96), *W* = marginal error of 5% (*w*=0.05), and *P* = the proportion of chronic oral medication use assumed to be 0.5 (50%); this is because no appropriate prior study was done in the study area regarding chronic oral medication based on the best of literature search done.

(1) $$\begin{array}{c} n=\frac{{\left(1.96\right)}^{2}\cdot \left(0.5\right)\left(0.5\right)}{{\left(0.05\right)}^{2}}=384. \end{array}$$

Considering the possible nonrespondent 5%, the final sample size was 402. Thus, 402 patients were equally allocated for 19 pharmacies and then the participants from each pharmacy were consecutively included in the study based on the following inclusion and exclusion criteria.

### 2.3. Inclusion and Exclusion Criteria

Patients were eligible to participate in the study if they had been taking oral medication at least once daily for a minimum of three consecutive months prior to recruitment into the study. All participants were older than 18 years. Patients who were not from Gondar, had cognitive or perceptual problems, failed to communicate, and were taking any nonprescription medications were excluded from this study.

### 2.4. Data Collection and Management

The data were collected through face-to-face interviews using a structured validated questionnaire. Fifth-year pharmacy students at each pharmacy gathered the data during their two-week experiential community pharmacy clerkship program. Data collectors and a supervisor were trained for two days by the principal investigators to ensure the quality of data collection. The questionnaire was pretested on 20 randomly selected participants. The reliability of the medication adherence questionnaire was measured and Cronbach's alpha value was 0.873. In addition, the knowledge questionnaire was evaluated using face validation and approved by two senior clinical pharmacists and researchers.

Patient details such as age (<65 years old and ≥65 years old), gender, education level (primary school means a school that includes grades 1–8, high school education means a school that includes grades 9 or 10 through 12, and above means college and university education), and duration of medication use (<1 year and >1 year) were used from patient data recorded during the face-to-face interviews at the community pharmacies. Education levels were classified as primary school and high school degrees. Patient drugs were classified according to the Anatomical Therapeutic Chemical Classification System (ATC).

Medication knowledge questionnaires were adapted from studies by McPherson et al. [22] and Okuyan et al. [23]. Patient knowledge was evaluated through a validated seven-item yes/no closed questionnaire type such as the following: Q1: correct if the participant states either generic or brand name and incorrect if the participant does not know, Q2: correct if the participant can state medication's exact working mechanism or correctly states the reason for the administration of medication and incorrect if the participant does not know, Q3: correct if the participant can correctly describe the administration method for this medication (e.g., tablet; swallowing the whole tablet with plenty of water) and incorrect if the participant does not know, Q4: correct if the participant correctly describes when to take this medication (i.e., on an empty stomach) and incorrect if the participant does not know, Question 5: correct if the participant can state the medication side effects, including those not experienced by the patient and incorrect if the participant does not know, Q6: correct if the participant states they would call their physician/pharmacist or stop taking the medication or other self-management intervention methods when faced with side effects and incorrect if the participant does not know, and Q7: correct if the participant says he or she never forgets a dose, he or she takes the next scheduled dose, or he or she calls the physician or pharmacist and incorrect if the participant does not know or declares he or she doubles up on doses. The number of correct responses was used to assess the total medication knowledge score. A score of 1 will be given for each question if correctly answered and 0 if not answered. Extra one point was granted to each participant if the exact mechanism of their medication was stated correctly. The total medication knowledge score was calculated without question 7. Therefore, the total medication knowledge score was evaluated out of seven from a total of six questions in the questionnaire. A score ≥5 was considered to be as good knowledge.

Medication adherence was assessed using the Adherence in Chronic Diseases Scale (ACDS) [24]. The questionnaire consists of 7 questions. Questions 1–5 concern the patient's behavior related to medication while questions 6 and 7 concern the physician-patient relationship. Based on the answer, each item of the scale is awarded 0–4 points. The sum of the total medication adherence score ranged from 0 to 28. The patient was classified as high adherence to treatment with a total score >26 points whereas scores of 21–26 and <21 points, respectively, correspond to medium adherence and low adherence. This questionnaire is available free of charge on the website of the Department of Health Promotion, Collegium Medicum, Nicolaus Copernicus University, Poland [24].

### 2.5. Statistical Analysis

The collected data was analyzed using Statistical Package for Social Sciences (SPSS) 22.0 (SPSS Inc., Chicago, IL, USA). The mean with standard deviations (SDs), frequencies, percentages, and odds ratios were evaluated using chi-square tests. The Mann–Whitney *U* test was applied to examine group differences in mean knowledge and adherence scores. Bivariate analysis was carried out to evaluate the crude effect of each independent variable on the different study outcomes. Variables with a *p* value less than or equal to 0.25 in the bivariate analysis were included in multivariate logistic regression models. Since the medication knowledge and adherence scores were not normally distributed using the Shapiro–Wilk test (*p* < 0.001), the relationship between the two scores was assessed using Spearman rank-order correlation. In all tests, results were considered significant if *p* < 0.05.

### 2.6. Ethical Clearance

This study was conducted after ethical clearance was obtained from the research and ethics review committees of the School of Pharmacy, University of Gondar, College of Medicine and Health Sciences, Gondar, Ethiopia. The permission to collect data was obtained after official letters were approved by the head of community pharmacies. All participants in the study were asked about their willingness to participate in the study. Information about the objective and contents of the study, as well as their right to refuse before any data collection, was given to all participants.

## 3. Result

A total of 402 patients who were with chronic diseases and use oral medications were selected from 19 randomly selected community pharmacies across Gondar City. The mean age of the study participants was 44.7 ± 16.9 years (range: 18–86 years). Two hundred and fourteen (53.2%) were males, 84.8% were aged <65 years, 60.7% had a high school education or above, and 86.6% had been using oral chronic medications for more than one year (Table 1).

**Figure 1:**
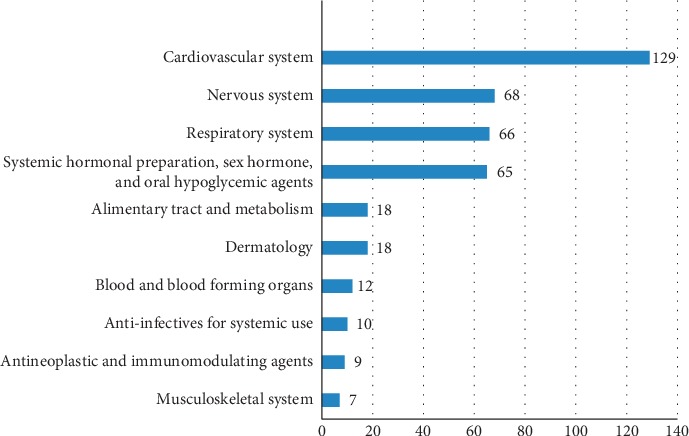
The categories of medications according to the ATC classification system.

The most frequently consumed oral medications based on their ATC classification were for the cardiovascular system (*n* = 129, 32%), nervous system (*n* = 68, 16.9%), and respiratory system (*n* = 66, 16.4%) (Figure 1).

In the assessment of medication knowledge, a majority of the participants expressed that they did not know “how to take medications” (80.6%), were “missing the doses” (54.5%), and did not know “what to do if side effects occur with medications” (53.5%). In addition, 77.6% knew how to take medications, 54.2% knew the reasons for taking the medications, and 51.2% correctly listed the name of their current medications. However, only 5.7% of the patients were able to explain the exact mechanism of action of their medications in general terms (Table 2).

The mean knowledge score is 3.71 ± 0.12 out of 7, and the average medication adherence score is 23.75 out of 28 of the respondents. 63 (15.7%) respondents obtained high ACDS scores (>26 points); 95 (23.6%), intermediate scores (21–26 points); and 244 (60.7%), low scores (<21 points) as shown in Table 3. A significant correlation was noticed between sociodemographic factors and medication knowledge and adherence scores. Although the participants demonstrated not good medication knowledge, sex, and adherence scores, patients with high school degrees (4.25 ± 0.08) and younger than 65 years (3.78 ± 0.08) showed better medication knowledge than their counterparts. In addition, no differences in medication knowledge were noticed between sex and duration of the treatment. However, having not good medication knowledge was noticed across all the study participants (Table 3). In addition, females, the less educated, and those who do not have good medication knowledge were found to have a significantly higher medication adherence score than their counterparts.

Binary logistic regression analysis revealed that the patients with high school and above education were more likely to have good medication knowledge (OR = 5.350, 95% CI 3.231–8.857, *p* < 0.001). However, gender, age, and the use of medications for more than one year did not show any significant correlation with good medication knowledge (Table 4).

Similarly, good medication knowledge scores were significantly associated with medication adherence (AOR = 10.300 95% CI = [6.16517.209]; *p* < 0.001). Male patients were also more likely to be adherent to their medication (OR = 1.936, 95% CI = 1.154–3.249, *p*=0.012) (Table 5).

## 4. Discussion

This study demonstrated the extent of oral medication knowledge and adherence among chronic disease patients attending community pharmacies in Gondar City, Ethiopia. We used the validated McPherson et al.'s medication knowledge scale [22], and Adherence in Chronic Diseases Scale (ACDS) [24] helped us to investigate possible associations between the level of knowledge and adherence among Ethiopian chronic disease patients. We assessed not only medication adherence but also its relationship with several sociodemographic factors and we strongly believe that our findings may contribute a greater understanding of this issue in this population.

Through this investigation, it is confirmed that knowledge about chronic oral medications was not good and most of the patients were not adherent to the medications. When knowledge about the use of medications was evaluated as a score out of 7, 60.4% of the chronic patients were found to have modest knowledge with a score of less than or equal to 4. This is in line with the Okuyan et al. study where 64.5% possess optimal knowledge about their medications [23]. Moreover, only a small fraction of patients knew the exact working mechanism of the drugs that they were taking. High school and above education was a predictor of good medication knowledge. Similar findings were noticed among the Jordanian chronic disease patients and Saudi Arabian patients visiting King Abdulaziz Medical Center and in the Sanchez-Romero et al. study conducted on Spanish patients attending community pharmacies [25–27]. This indicates that a lack of knowledge about chronic medications remains an important problem in many countries. However, in Ethiopia, community pharmacists did not consider providing pharmaceutical care activities such as medication counseling and monitoring and describing the long-term benefits of medication adherence to chronic disease patients to be a part of their routine practice. Pharmacists in Ethiopia could take a new initiative to provide extensive pharmaceutical care services to improve medication knowledge and adherence, especially for chronically ill patients.

Our results showed that more than 60% of the patients had low adherence and had a difficulty remembering their medications. These results were consistent with previous studies [28, 29]. Low education was identified as a risk factor for the lack of medication-related knowledge; similar findings were highlighted in other studies [4, 26, 27, 30]. Logistic regressions showed that knowledgeable patients are ten times more likely to have high medication adherence. Specifically, patients having knowledge about the name of their medications, how to take them, what to do if a dose was missed, and what to do if side effects occurred were shown to have a significant association with greater medication adherence.

To our knowledge, this is the first study that assessed the relationship between knowledge about oral chronic medications and medication adherence in low-income Sub-Saharan African countries such as Ethiopia. Several studies have investigated the factors associated with adherence to chronic medication [31–33]. All of these studies focused on adherence to medications for specific diseases such as diabetes mellitus or hypertension [34–36], and they all highlighted medication nonadherence as contributing to poorer control of the chronic disease. We included patients with any type of chronic diseases, making our results more generalizable to other diseases. In addition, we used validated, reliable tools for the assessment of medication knowledge and medication adherence that covered both aspects simultaneously for all types of chronic diseases. Further studies focusing on pharmacist-led educational interventions on patient medication knowledge and its association with their medication adherence could be investigated.

There were some limitations to the present study that need to be considered. First, it is a cross-sectional study design, whereby claims about the directionality of the causal relationship between the dependent and independent variables cannot be verified. In addition, we used convenience sampling, which can lead to the underrepresentation or overrepresentation of particular groups within the sample. Second, the use of self-reporting can potentially lead to recall bias by the respondents, which should be taken into consideration while interpreting the results. Moreover, we obtained and evaluated data of oral medications used by chronically ill patients for at least 3 months. Third, the administration of the questionnaire through face-to-face interviews by the trained pharmacy students in the community pharmacy settings may increase the respondents' inclination to give socially acceptable answers. To prevent this, we ensured the confidentiality and privacy of the participants.

## 5. Conclusion

The results of this study found that the overall knowledge of and adherence to oral chronic medications was not good in our study population. Good medication knowledge and female patients were significant predictors for medication adherence. These findings emphasize the need for pharmacists-led medication counseling to improve the information level in people affected by chronic diseases and to improve the adherence to oral chronic medications.

## Figures and Tables

**Table 1:** Sociodemographic details of study population (*N* = 402).

Characteristics	*N* (%)
Sex	
Male	214 (53.2)
Female	188 (46.8)

Age	
Less than 65 years	341 (84.8)
≥65 years	61 (15.2)

Education	
Primary school or below	158 (39.3)
High school or above	244 (60.7)

Medication use	
<one year	54 (13.4)
≥one year	348 (86.6)

**Table 2:** Medication knowledge among chronic disease patients (*N* = 402).

Statements		Score		
		1	0	2^*∗*^
1	Can you list the name of medications you are currently taking?	206 (51.2%)	196 (48.8%)	—
2	Can you tell me why you are taking this medication?	218 (54.2%)	161 (40%)	23 (5.7%)
3	Do you know how to take medication?	78 (19.4%)	324 (80.6%)	—
4	Do you know when to take your medicine?	312 (77.6%)	90 (22.4%)	—
5	Do you know the possible side effects of your medicines?	199 (49.5%)	203 (50.5%)	—
6	Do you know what to do if your medication side effects occur?	187 (46.5%)	215 (53.5%)	—
7	Do you know what to do if you miss a dose of your medicines?	183 (45.5%)	219 (54.5%)	—

1 = correctly answered; 0 = does not know; ^*∗*^2 *=* participant stated medication's exact working mechanism in general terms (^*∗*^only for question number 2).

**Table 3:** The distribution of participants according to demographic variables and the results of medication knowledge scores and medication adherence scale.

	Mean medication knowledge scores		Mean medication adherence score	
	Mean ± SEM	*p*	Mean ± SEM	*p*
Age		0.024		NS
<65 years old	3.78 ± 0.08		23.61 ± 0.06	
≥65 years old	3.33 ± 0.18		23.84 ± 0.14	

Gender		NS		0.036
Male	3.71 ± 0.10		22.53 ± 0.07	
Female	3.72 ± 0.11		21.77 ± 0.08	

Education status		<0.001		<0.001
<high school degree	2.87 ± 0.11		25.87 ± 0.09	
≥high school degree	4.25 ± 0.08		24.09 ± 0.06	

Duration of medication utilization		NS		NS
<1 year	4.00 ± 0.19		21.67 ± 0.22	
≥1 year	3.67 ± 0.08		21.64 ± 0.05	

Good medication knowledge				<0.001
Yes (*n* = 154)	—		25.1 ± 0.89	
No (*n* = 248)	—		23.94 ± 0.90	

SEM = standard error.

**Table 4:** Determinants of good medication knowledge.

Variable		Number of patients having good medication knowledge		COR	AOR at 95% CI	*p* value
		Yes	No			
Gender	Male	81	133	1	1	0.082
	Female	73	115	1.042	1.492 [0.950–2.341]	
Age	≥65 years old	16	45	1	1	0.427
	<65 years old	138	203	1.912	1.307 [0.675–2.532]	
Education	<High school degree	28	130	1	1	<0.001^*∗*^
	≥High school degree	126	118	4.958	5.350 [3.231–8.857]	
Duration of medication utilization	<1 year	20	34	1	1	0.442
	≥1 year	134	214	1.064	1.281 [0.682–2.407]	

Good medication knowledge: patients with ≥5 medication knowledge score. COR = crude odds ratio; AOR = adjusted odds ratio; 95% CI = confidence interval; ^*∗*^significant at *p* < 0.05.

**Table 5:** Determinants of medication adherence.

Variable		Number of patients being adherent		COR	AOR at 95% CI	*p* value
		Yes	No			
Gender	Female	63	125	1	1	0.012^*∗*^
	Male	95	119	1.584	1.936 [1.154–3.249]	
Age	≥65 years old	16	45	1	1	0.209
	<65 years old	142	199	2.007	1.605 [0.767–3.360]	
Education	<High degree	34	124	1	1	0.063
	≥High school degree	124	120	3.769	1.681 [0.972–2.907]	
Duration of medication utilization	≥1 year	133	215	1	1	0.095
	<1 year	25	29	1.394	1.820 [0.901–3.677]	
Good medication knowledge	No	47	201	1		<0.001^*∗*^
	Yes	111	43	11.040	10.300[6.16517.209]	

COR = crude odds ratio; AOR = adjusted odds ratio; 95% CI = confidence interval; ^*∗*^: significant at *p* < 0.05.

## Data Availability

All available data can be obtained by contacting the corresponding author.

## References

[1] Stirratt M. J., Dunbar-Jacob J., Crane H. M. (2015). Self-report measures of medication adherence behavior: recommendations on optimal use. *Translational Behavioral Medicine*.

[2] Zeber J. E., Manias E., Williams A. F. (2013). A systematic literature review of psychosocial and behavioral factors associated with initial medication adherence: a report of the ISPOR medication adherence & persistence special interest group. *Value in Health*.

[3] Buszko K., Obońska K., Michalski P. (2016). The adherence scale in chronic diseases (ASCD). The power of knowledge: the key to successful patient—health care provider cooperation. *Medical Research Journal*.

[4] Najjar A., Amro Y., Kitaneh I. (2015). Knowledge and adherence to medications among Palestinian geriatrics living with chronic diseases in the WestBank and East Jerusalem. *PLoS One*.

[5] Ho P. M., Bryson C. L., Rumsfeld J. S. (2009). Medication adherence. *Circulation*.

[6] Altin S. V., Finke I., Kautz-Freimuth S., Stock S. (2014). The evolution of health literacy assessment tools: a systematic review. *BMC Public Health*.

[7] Giardini A., Martin M. T., Cahir C. (2016). Towards appropriate criteria in medication adherence assessment in older persons: position paper. *Aging Clinical and Experimental Research*.

[8] World Health Organization (2002). *The World Health Report 2002: Reducing Risks, Promoting Healthy Life*.

[9] Lavsa S. M., Holzworth A., Ansani N. T. (2011). Selection of a validated scale for measuring medication adherence. *Journal of the American Pharmacists Association*.

[10] Svarstad B. L., Chewning B. A., Sleath B. L., Claesson C. (1999). The brief medication questionnaire: a tool for screening patient adherence and barriers to adherence. *Patient Education and Counseling*.

[11] McDonnell P. J., Jacobs M. R., Monsanto H. A., Kaiser J.-M. (2002). Hospital admissions resulting from preventable adverse drug reactions. *Annals of Pharmacotherapy*.

[12] Ho P. M., Magid D. J., Shetterly S. M. (2008). Medication non-adherence is associated with a broad range of adverse outcomes in patients with coronary artery disease. *American Heart Journal*.

[13] Rasmussen J. N., Chong A., Alter D. A. (2007). Relationship between adherence to evidence-based pharmacotherapy and long-term mortality after acute myocardial infarction. *JAMA*.

[14] Federal Ministry of Health (FMOH) and Global Health (2018). *Supply Chain-Procurement and Supply Management (GHSC-PSM)*.

[15] Taylor K., Harding G. (2005). *Pharmacy Practice*.

[16] Erku D. A., Mekurie A. B., Surrur A., Gebresillassie B. M. (2016). Extent of dispensing prescription-only medications without a prescription in community drug retail outlets in Addis Ababa, Ethiopia: a simulated-patient study. *Drug, Healthcare and Patient Safety*.

[17] Drug Administration and Control Authority of Ethiopia (2012). *Manual for Good Dispensing Practice*.

[18] Wabe N. T., Raju N., Angamo M. T. (2011). Knowledge, attitude and practice of patient medication counseling among drug dispensers in North West Ethiopia. *Journal of Applied Pharmaceutical Science*.

[19] Nigussie W. (2014). Patient counseling at dispensing of medicines in health care facility outpatient pharmacies of Bahir Dar city, Northwest Ethiopia. *Science Journal of Public Health*.

[20] Ngoh L. N. (2009). Health literacy: a barrier to pharmacist-patient communication and medication adherence. *Pharmacy Today*.

[21] (2016). *Health Sector Development Program IV*.

[22] McPherson M. L., Smith S. W., Powers A., Zuckerman I. H. (2008). Association between diabetes patients’ knowledge about medications and their blood glucose control. *Research in Social and Administrative Pharmacy*.

[23] Okuyan B., Sancar M., Izzettin F. V. (2013). Assessment of medication knowledge and adherence among patients under oral chronic medication treatment in community pharmacy settings. *Pharmacoepidemiology and Drug Safety*.

[24] Kubica A., Kosobucka A., Michalski P. (2017). The adherence in chronic diseases scale—a new tool to monitor implementation of a treatment plan. *Folia Cardiologica*.

[25] Awwad O., Akour A., Al-Muhaissen S., Morisky D. (2015). The influence of patients’ knowledge on adherence to their chronic medications: a cross-sectional study in Jordan. *International Journal of Clinical Pharmacy*.

[26] Alkatheri A., Albekairy A. (2013). Does the patients’ educational level and previous counselling affect their medication knowledge?. *Annals of Thoracic Medicine*.

[27] Romero-Sanchez J., Garcia-Cardenas V., Abaurre R., Martínez-Martínez F., Garcia-Delgado P. (2016). Prevalence and predictors of inadequate patient medication knowledge. *Journal of Evaluation in Clinical Practice*.

[28] Napolitano F., Napolitano P., Angelillo I. F. (2016). Medication adherence among patients with chronic conditions in Italy. *The European Journal of Public Health*.

[29] Al-Qazaz H. K., Sulaiman S. A., Hassali M. A. (2011). Diabetes knowledge, medication adherence and glycemic control among patients with type 2 diabetes. *International Journal of Clinical Pharmacy*.

[30] Cumbler E., Wald H., Kutner J. (2010). Lack of patient knowledge regarding hospital medications. *Journal of Hospital Medicine*.

[31] Wabe N., Angamo M., Hussein S. (2011). Medication adherence in diabetes mellitus and self management practices among type-2 diabetics in Ethiopia. *North American Journal of Medical Sciences*.

[32] Ambaw A. D., Alemie G. A., Yohannes S. M. W., Mengesha Z. B. (2012). Adherence to antihypertensive treatment and associated factors among patients on follow up at University of Gondar Hospital, Northwest Ethiopia. *BMC Public Health*.

[33] Kassahun T., Gesesew H., Mwanri L., Eshetie T. (2016). Diabetes related knowledge, self-care behaviours and adherence to medications among diabetic patients in Southwest Ethiopia: a cross-sectional survey. *BMC EndocrDisord*.

[34] Mekonnen H. S., Gebrie M. H., Eyasu K. H., Gelagay A. A. (2017). Drug adherence for antihypertensive medications and its determinants among adult hypertensive patients attending in chronic clinics of referral hospitals in Northwest Ethiopia. *BMC PharmacolToxicol*.

[35] Abebaw M., Messele A., Hailu M., Zewdu F. (2016). Adherence and associated factors towards antidiabetic medication among type II diabetic patients on follow-up at university of Gondar hospital, Northwest Ethiopia. *Advances in Nursing*.

[36] Kassahun A., Gashe F., Mulisa E., Rike W. A. (2016). Non-adherence and factors affecting adherence of diabetic patients to anti-diabetic medication in Assela General Hospital, Oromia Region, Ethiopia. *Journal of Pharmacy and Bioallied Sciences*.

